# Polyvinyl alcohol cryogel phantoms of biological tissues for wideband operation at microwave frequencies

**DOI:** 10.1371/journal.pone.0219997

**Published:** 2019-07-25

**Authors:** Natalia Arteaga-Marrero, Enrique Villa, Javier González-Fernández, Yolanda Martín, Juan Ruiz-Alzola

**Affiliations:** 1 IACTec Medical Technology Group, Instituto de Astrofísica de Canarias (IAC), La Laguna, Santa Cruz de Tenerife, Spain; 2 Departamento de Ingeniería Biomédica. Instituto Tecnológico de Canarias (ITC), Santa Cruz de Tenerife, Santa Cruz de Tenerife, Spain; 3 Departamento de Señales y Comunicaciones. Instituto Universitario de Investigación Biomédica y Sanitaria (IUIBS). Universidad de Las Palmas de Gran Canaria, Las Palmas de Gran Canaria, Las Palmas, Spain; New York University School of Medicine, UNITED STATES

## Abstract

The aim of this work is to provide a methodology to model the dielectric properties of human tissues based on phantoms prepared with an aqueous solution, in a semi-solid form, by using off-the-shelf components. Polyvinyl alcohol cryogel (PVA-C) has been employed as a novel gelling agent in the fabrication of phantoms for microwave applications in a wide frequency range, from 500 MHz to 20 GHz. Agar-based and deionized water phantoms have also been manufactured for comparison purposes. Mathematical models dependent on frequency and sucrose concentration are proposed to obtain the complex permittivity of the desired mimicked tissues. These models have been validated in the referred bandwidth showing a good agreement to experimental data for different sucrose concentrations. The PVA-C model provides a great performance as compared to agar, increasing the shelf-life of the phantoms and improving their consistency for contact-required devices. In addition, the feasibility of fabricating a multilayer phantom has been demonstrated with a two-layer phantom that exhibits a clear interface between each layer and its properties. Thus, the use of PVA-C extends the option for producing complex multilayer and multimodal phantoms.

## Introduction

Recently, a growing interest and research on the interaction of electromagnetic fields with biological tissues has been observed, particularly at microwave frequencies [1], which has been mainly motivated by an increased use of devices featuring electromagnetic radiation within this band. The application of such devices in imaging raised interest because microwave technology offers a suitable trade-off between depth penetration, image resolution, and contrast between dielectric properties of healthy and malignant tissues [2]. In order to passively measure temperature patterns in depth, a microwave radiometer system is being developed at our facilities. Since ethical and legal issues prevent human testing, artificially produced materials that mimic human body tissues, known as phantoms, are required to enable the system characterization.

The fabrication of phantoms in the microwave spectrum covers a wide range of applications. For magnetic resonance imaging, phantoms are employed in coil development at high magnetic fields, quality assurance, system tests, and pulse sequence evaluation for new imaging techniques [3–5]. Moreover, for cancer diagnosis systems, phantoms mimicking breast as well as brain are fabricated to test and validate the developed systems [2, 6, 7]. In addition, body area networks (BANs) operating in the 2.4 GHz frequency band require the use of phantoms to characterize the influence of the electromagnetic radiation in humans’ health [8, 9].

The suitability of a phantom depends on the frequency and the targeted tissue [8]. Ideally, phantoms should be composed of low-cost materials, easy to obtain (off-the-shelf) and stable over long periods of storage time [6]. Water-soluble ingredients are preferred to generate homogeneous solutions to avoid complex mixing procedures or toxic additives [3]. Usually, deionized water is employed as the solvent. Phantoms should replicate the features of body tissues, in terms of complex permittivity. Sucrose is added to control its real part ($\varepsilon_{r}^{{}^{\prime }}$), known as dielectric constant or permittivity; and sodium chloride (NaCl) to modulate its imaginary part ($\varepsilon_{r}^{{}^{\prime \prime }}$), known as the dielectric loss, directly related to conductivity. The dielectric constant decreases with an increment in sucrose concentration whereas the dielectric loss increases with an increment in sodium chloride, although a non-linear dependence is observed [8]. A similar trend of non-linearity with frequency is exhibited by biological tissues, which behave as a dielectric with losses. Conservation agents are used to preserve the mixture, such as sunflower seed oil, formaldehyde, formalin or benzoic acid [2, 3, 10]. When a semisolid phantom is required, the solution is combined with a gelling agent, being agar the most commonly used, which also reduces heat diffusivity as opposed to liquid phantoms [3].

Currently, a significant issue with the phantom fabrication is the lack of long-term stability. Properties vary in time due to decomposition and dry out of the constituent materials, thus repeatability of measurements over time cannot be ensured [2, 11, 12]. In fact, a drawback of using agar as thickening material is the exudation of water from the phantom, which may influence the measurement of the dielectric permittivity [7]. Furthermore, since agar is a natural product, phantom properties may differ depending on the batch, oppositely to polymer synthesis that is adequately standardized [4]. The addition of antimicrobial agents may extend the availability of the phantom; however, limited shelf-life is expected from a few days up to four months [5, 6, 13]. Furthermore, most phantoms are designed to work in narrow bandwidths, and operational phantoms in a large bandwidth are scarce [9].

Another issue is that phantoms are usually liquid. This is a serious limitation for the creation of realistic and heterogeneous multilayer phantoms, which mimic the different tissues that human organs consist of. Another difficulty arises from the solvent diffusion that occurs when two materials with different concentrations of gelling agent are placed in direct contact [1]. Additionally, the interaction with some microwave systems could be difficult when using contact antennas, in which physical contact with the mimicking surfaces is required.

Polyvinyl alcohol cryogel (PVA-C) is proposed as an alternative gelling agent in the fabrication of phantoms. This hydrophilic and biocompatible polymer [14] transforms into a solid hydrogel by physical crosslinking, and exhibits mechanical properties similar to those of biological tissues [15]. PVA-C can be molded easily and is not toxic [16]. Solidification is achieved by gelation using freeze (-20°C) and thaw (+20°C) cycles that provide high strength to the gel [14] and modify the electrical properties of the phantom [17]. PVA-C presents high optical transparency and can reproduce elasticity and viscosity similar to soft tissues [11], keeping its wetness over an extended period of time [16]. PVA-C has been previously used in research and development of endovascular devices, reproducing soft tissue models like arteries [16, 18], as well as to fabricate phantoms for ultrasonography [11]. PVA-C provides a unique opportunity to manufacture multilayer and multimodal phantoms. However, to the authors’ knowledge, it has not been previously used in the fabrication of phantoms for microwave applications.

In this work, PVA-C has been employed as a novel and alternative gelling agent in the fabrication of custom phantoms for microwave applications in a large frequency band (from 500 MHz to 20 GHz). A set of phantoms based on aqueous solutions was manufactured with varying concentration of sucrose (0%, 15%, 30%, 45% and 60%). For comparison purposes, a similar set of phantoms was also produced employing the commonly used agar, as well as a liquid model without gelling agent. For each of these sets of phantoms (PVA-C, agar and deionized water), a mathematical model dependent on frequency and sucrose concentration was fitted to enable the adjustment of the complex permittivity of the desired mimicked tissue. These fitted models were validated by comparison to the theoretical Cole-Cole model [8, 19–21]. Thus, we propose a full methodology for the fabrication of custom off-the-shelf phantoms with specific dielectric properties in the mentioned frequency band. In addition, a two-layer phantom has been manufactured to demonstrate the capabilities of PVA-C for multilayer phantom fabrication.

## Materials and methods

### Phantom preparation

Three sets of phantoms were fabricated using two gelling agents: PVA-C (99% hydrolyzed, molecular weight 89000-98000, Sigma Aldrich) and agar for microbiology (Sigma Aldrich), and a third one without gelling agent. They were all composed of deionized water, varying concentrations of sucrose in the form of table sugar (99%) and benzoic acid (Sigma Aldrich) for preservation purposes.

The protocol to prepare the phantoms consisted of measuring the ingredients and mixing them in a container. Phantoms were created with the following concentrations of sucrose (percentage in weight/volume): 0%, 15%, 30%, 45% and 60% at a fixed concentration of benzoic acid (0.1%) [3]. The phantoms that contained a gelling agent were heated and magnetically stirred until the solutes have completely dissolved, resulting in an uniform solution. Regarding the PVA-C, the solvent consisted of a mixture of deionized water and PVA-C at a concentration of 15% [17]. Heat was required to dissolve the PVA-C and the benzoic acid by raising the temperature to approximately 70°C. In the case of the agar-based phantoms, agar was added until a 3% concentration using deionized water as solvent, since a suitable solidification was not achieved for lower concentration values. The temperature required to activate the agar was 70°C, but the solution was heated up to nearly 80°C to achieve the expected consistency.

Subsequently, each mixture was poured in labelled plastic containers (100 ml) in a slow and controlled manner, minimizing the amount of air bubbles. The phantoms were naturally cooled down at room temperature. Afterwards, the agar-based phantoms were introduced into a fridge for conservation purposes. The PVA-C-based phantoms required freeze and thaw cycles to achieve the cross-linking of the polymer. Thus, the phantoms were introduced in the freezer overnight and, then, thawed and stored in the fridge until measurement. A suitable consistency was observed after a single freeze and thaw cycle. An increment in the number of cycles affects the dielectric properties of the material, decreasing the conductivity of the phantom, as reported for lower frequencies [17]. Therefore, one cycle was considered for the manufactured phantoms.

### Characterization method

The procedure for the experimental characterization of the phantoms provides their dielectric features, using a well-known measurement protocol based on the use of an open-ended coaxial probe [22, 23]. The measurement setup consists of a vector network analyzer (FieldFox Handheld Microwave Analyzer up to 26.5 GHz, model N9918A, Keysight Technologies) and a coaxial probe (Performance Probe, model 85070E, Keysight Technologies). These devices are managed by a laptop, which also enables data processing. This setup measures the input reflection coefficient of the phantom under measurement, and the associated software platform translates it into the complex permittivity, with its real and imaginary parts.

The Performance Probe used in the measurements covers the frequency range up to 50 GHz, and it is configured with a 2.4 mm connector interface. However, the maximum frequency of the setup is limited by the maximum operation frequency of the analyzer. Real part of relative permittivities lower than 100 can be managed with this probe, and it is not recommended for low-loss materials (loss tangent below 0.5) [24, 25].

The system is calibrated using a three-standard procedure: an open circuit, a short circuit and deionized water. The physical temperature of the water was also required as an input for the calibration (23°C). Dimethyl sulfoxide (DMSO, Sigma Aldrich) and methanol (Sigma Aldrich) were employed to validate the measurement procedure. Accuracy was considered as the average percentage variation between the measured values and the models reported in the literature over the entire frequency range (from 500 MHz to 20 GHz) [13, 26, 27]. Repeatability was calculated as the standard deviation divided by the mean value over the entire frequency range [26–28]. DMSO dielectric measurements were compared to the Cole-Davidson model up to 20 GHz [26]. The accuracy in the measurements was within 4% and 7% for the real and imaginary parts of the complex permittivity ($\varepsilon_{r}^{{}^{\prime }}$ and $\varepsilon_{r}^{{}^{\prime \prime }}$), respectively, whereas the mean percentage of variation for repeated measurements were both within 0.5%. Regarding the methanol, $\varepsilon_{r}^{{}^{\prime }}$ and $\varepsilon_{r}^{{}^{\prime \prime }}$ measurements were compared to the Debye and Cole-Cole models which are well defined up to 5 GHz [26]. In this range, measurement accuracy was found within 4% and 10% for $\varepsilon_{r}^{{}^{\prime }}$ and $\varepsilon_{r}^{{}^{\prime \prime }}$, respectively; while repeatabilities were both within 0.2%. The measured $\varepsilon_{r}^{{}^{\prime }}$ at 2 and 3 GHz were 44.351 ± 0.021, 41.715 ± 0.009 and 24.988 ± 0.013, 19.828 ± 0.018 for DMSO and methanol, respectively. Reported values in the literature [26] at these frequencies for both materials were 44.596 and 41.808 for DMSO whereas 24.881 and 19.259 for methanol.

**Fig 1:**
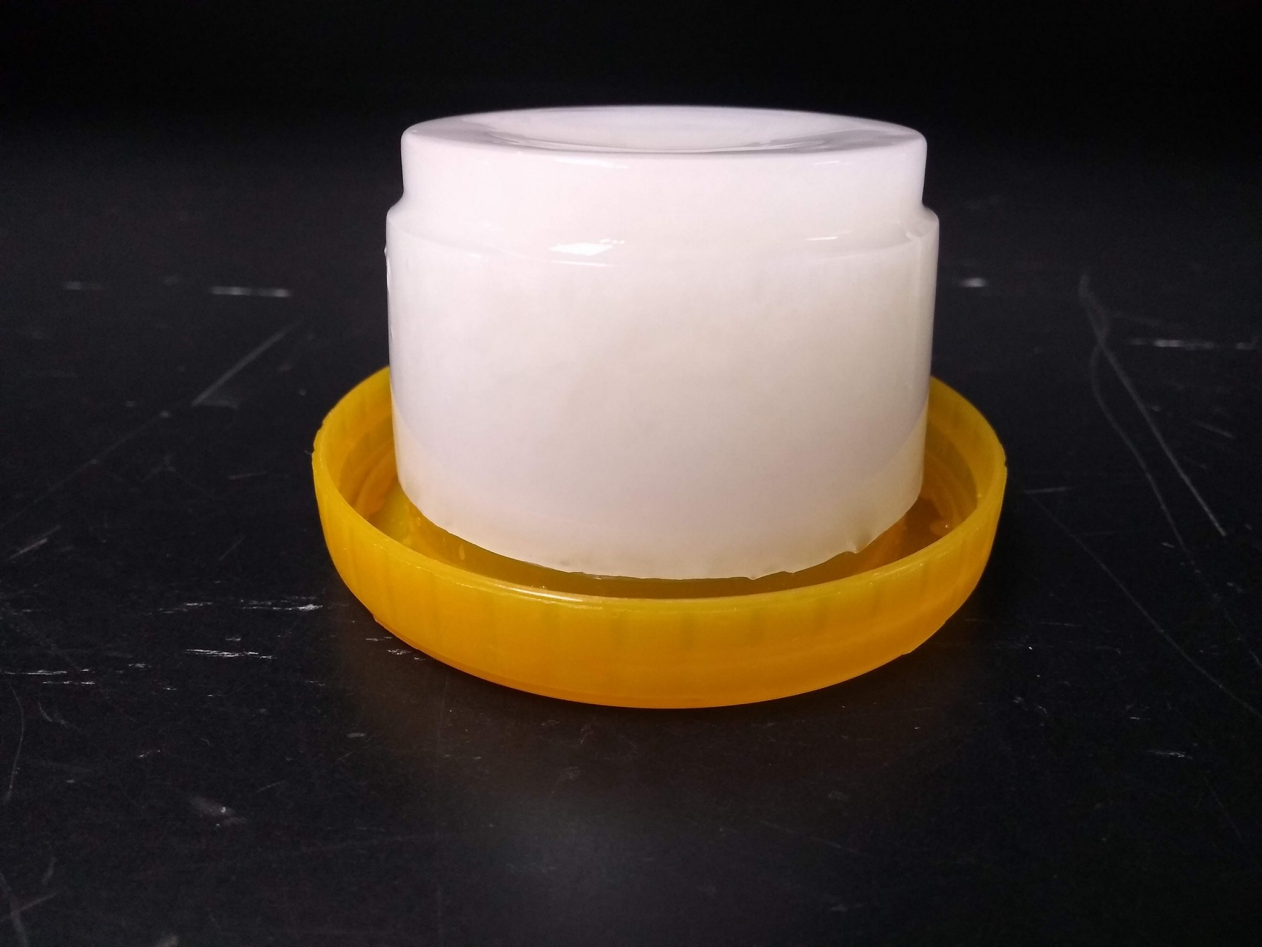
PVA-C phantom. PVA-C-based phantom with a sucrose concentration of 15%.

**Fig 2:**
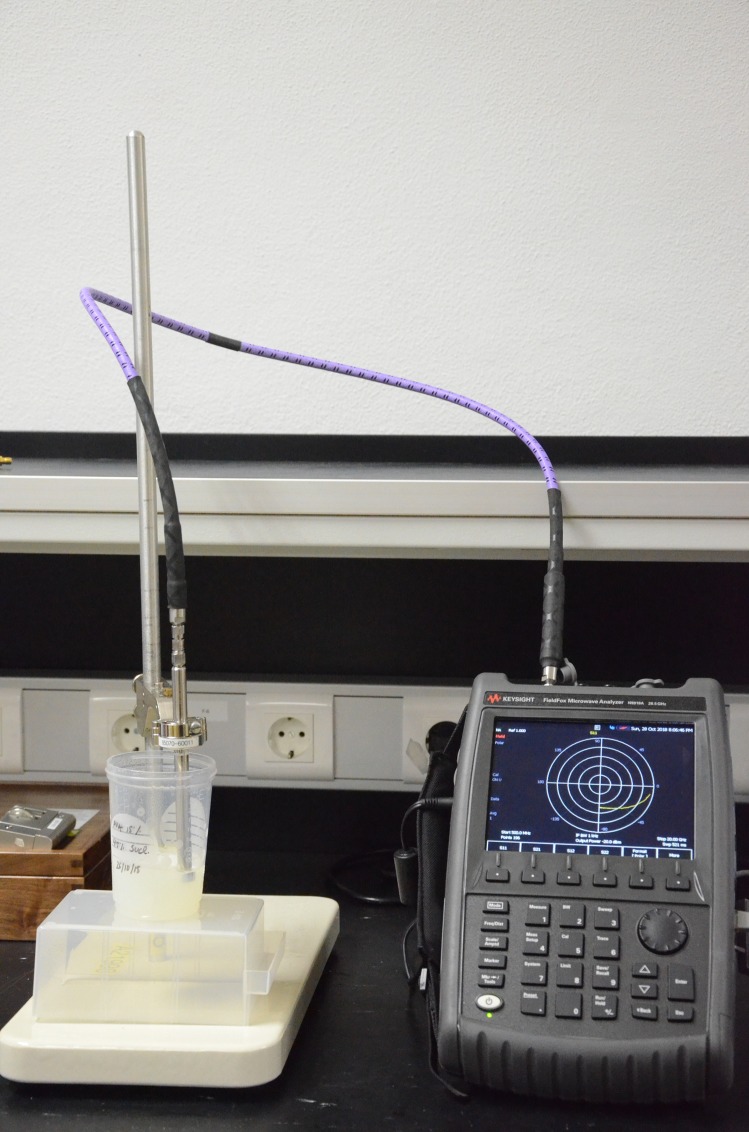
Measurement procedure. Setup to measure the dielectric properties of each fabricated phantom.

Measurements were acquired between 0.5 and 20 GHz using 196 linearly spaced frequency points. The data acquisition consisted of ten consecutive measurements, which were subsequently averaged to provide the daily measurement. Phantoms were measured at room temperature (23°C) by immersing the dielectric probe in the phantom body. In case of PVA-C, the probe was positioned in direct contact with the surface of the phantom. Deionized water was used between measurements to clean the probe. A PVA-C-based phantom with a sucrose concentration of 15% is shown in Fig 1, while Fig 2 shows the setup used to measure each fabricated phantom.

The homogeneity of the fabricated phantoms was assessed by averaging the dielectric properties, $\varepsilon_{r}^{{}^{\prime }}$ and $\varepsilon_{r}^{{}^{\prime \prime }}$, measured in five random spots. The measurement in each spot consisted of averaging ten consecutive acquisitions. Subsequently, the percentage of variation was calculated and averaged for all the frequency points. The mean percentages of variation for $\varepsilon_{r}^{{}^{\prime }}$ and $\varepsilon_{r}^{{}^{\prime \prime }}$ were below 2%, 0.5%, and 1%, for the PVA-C-, agar- and deionized water based phantoms, respectively.

### Model extraction

Mathematical models to characterize PVA-C, agar, and deionized water phantoms are proposed based on frequency and sucrose concentration. The fitted models provide a design methodology considering the real and imaginary parts of the dielectric relative permittivity, $\varepsilon_{r}^{{}^{\prime }}$ and $\varepsilon_{r}^{{}^{\prime \prime }}$, respectively. The procedure to obtain each model is to acquire experimental data ($\varepsilon_{r}^{{}^{\prime }}$, $\varepsilon_{r}^{{}^{\prime \prime }}$) for phantoms with different sucrose concentrations (from 0% up to 60% in steps of 15%) in the frequency band from 500 MHz to 20 GHz.

To this extent, third-order polynomials properly represent both the real and imaginary parts of the relative permittivity as a function of frequency, *f (GHz)*, expressed as:

$$\begin{array}{c} \varepsilon_{r}^{{}^{\prime }}=a_{\varepsilon^{{}^{\prime }}}\cdot f^{3}+b_{\varepsilon^{{}^{\prime }}}\cdot f^{2}+c_{\varepsilon^{{}^{\prime }}}\cdot f+d_{\varepsilon^{{}^{\prime }}} \end{array}$$ (1)

$$\begin{array}{c} \varepsilon_{r}^{{}^{\prime \prime }}=a_{\varepsilon^{{}^{\prime \prime }}}\cdot f^{3}+b_{\varepsilon^{{}^{\prime \prime }}}\cdot f^{2}+c_{\varepsilon^{{}^{\prime \prime }}}\cdot f+d_{\varepsilon^{{}^{\prime \prime }}} \end{array}$$ (2)

where *a*, *b*, *c*, and *d* are a set of fitting parameters dependent on sucrose concentration, *S_*c*_*, which are fitted to a quadratic function, following a similar approach as previous studies [8, 29]. Thus, Eqs (1) and (2) were transformed in terms of *S_*c*_* as follows:

$$\begin{array}{c} \begin{array}{c} \varepsilon_{r}^{{}^{\prime }}=\left[A_{\varepsilon^{{}^{\prime }}}^{{}^{\prime }}\cdot S_{c}^{2}+A_{\varepsilon^{{}^{\prime }}}^{{}^{\prime \prime }}\cdot S_{c}+A_{\varepsilon^{{}^{\prime }}}^{{}^{\prime \prime \prime }}\right]\cdot f^{3}+\left[B_{\varepsilon^{{}^{\prime }}}^{{}^{\prime }}\cdot S_{c}^{2}+B_{\varepsilon^{{}^{\prime }}}^{{}^{\prime \prime }}\cdot S_{c}+B_{\varepsilon^{{}^{\prime }}}^{{}^{\prime \prime \prime }}\right]\cdot f^{2}\\ +\left[C_{\varepsilon^{{}^{\prime }}}^{{}^{\prime }}\cdot S_{c}^{2}+C_{\varepsilon^{{}^{\prime }}}^{{}^{\prime \prime }}\cdot S_{c}+C_{\varepsilon^{{}^{\prime }}}^{{}^{\prime \prime \prime }}\right]\cdot f+\left[D_{\varepsilon^{{}^{\prime }}}^{{}^{\prime }}\cdot S_{c}^{2}+D_{\varepsilon^{{}^{\prime }}}^{{}^{\prime \prime }}\cdot S_{c}+D_{\varepsilon^{{}^{\prime }}}^{{}^{\prime \prime \prime }}\right] \end{array} \end{array}$$ (3)

$$\begin{array}{c} \begin{array}{c} \varepsilon_{r}^{{}^{\prime \prime }}=\left[A_{\varepsilon^{{}^{\prime \prime }}}^{{}^{\prime }}\cdot S_{c}^{2}+A_{\varepsilon^{{}^{\prime \prime }}}^{{}^{\prime \prime }}\cdot S_{c}+A_{\varepsilon^{{}^{\prime \prime }}}^{{}^{\prime \prime \prime }}\right]\cdot f^{3}+\left[B_{\varepsilon^{{}^{\prime \prime }}}^{{}^{\prime }}\cdot S_{c}^{2}+B_{\varepsilon^{{}^{\prime \prime }}}^{{}^{\prime \prime }}\cdot S_{c}+B_{\varepsilon^{{}^{\prime \prime }}}^{{}^{\prime \prime \prime }}\right]\cdot f^{2}\\ +\left[C_{\varepsilon^{{}^{\prime \prime }}}^{{}^{\prime }}\cdot S_{c}^{2}+C_{\varepsilon^{{}^{\prime \prime }}}^{{}^{\prime \prime }}\cdot S_{c}+C_{\varepsilon^{{}^{\prime \prime }}}^{{}^{\prime \prime \prime }}\right]\cdot f+\left[D_{\varepsilon^{{}^{\prime \prime }}}^{{}^{\prime }}\cdot S_{c}^{2}+D_{\varepsilon^{{}^{\prime \prime }}}^{{}^{\prime \prime }}\cdot S_{c}+D_{\varepsilon^{{}^{\prime \prime }}}^{{}^{\prime \prime \prime }}\right] \end{array} \end{array}$$ (4)

The dielectric conductivity can be derived from Eq (4) using the following expression [8, 30]:

$$\begin{array}{c} \sigma =2\cdot \pi \cdot f\cdot \varepsilon_{o}\cdot \varepsilon_{r}^{{}^{\prime \prime }} \end{array}$$ (5)

### Cole-Cole model

The Cole-Cole model [8, 19–21] has been commonly used to describe the experimental data for the dielectric constant, as a function of frequency, offering an accurate fit to biological tissues over a wide frequency range.

The validity of the single-pole Cole-Cole model has been previously investigated for the frequency bandwidth employed in this work, from 500 MHz to 20 GHz, concluding that increased accuracy is not observed with a two-pole or higher order model [21, 31]. Thus, the single-pole model, which is based in five parameters, is expressed as follows:

$$\begin{array}{c} \varepsilon_{r}=\varepsilon_{\infty }+\frac{\varepsilon_{s}-\varepsilon_{\infty }}{1+jw\tau^{\left(1-\alpha \right)}}+\frac{\sigma_{s}}{jw\varepsilon_{0}}=\varepsilon_{\infty }+\frac{\Delta \varepsilon }{1+jw\tau^{\left(1-\alpha \right)}}+\frac{\sigma_{s}}{jw\varepsilon_{0}} \end{array}$$ (6)

where *ε*_*s*_ is the static permittivity, *ε*_∞_ the high frequency permittivity limit, *τ* [s] the relaxation time, *σ*_*s*_[S/m] the static ionic conductivity, and an empirical parameter, *α*, that accounts for the observed broad distribution of relaxation time constants in tissues [31].

The real and imaginary parts of the relative permittivity have been fitted as an equation system [20]. Commonly, either *α* or *ε*_∞_, which depends on the tissue water content [21], are fixed in order to perform the fitting procedure [8, 29, 31]. However, no constraints were considered in this work and all the model parameters were freely varied.

## Results and discussion

### Model extraction

The measured multidimensional data depend on both the frequency and the sucrose concentration and describe the complex permittivity in the range from 500 MHz to 20 GHz for the different phantoms: PVA-C, agar, and deionized water.

**Fig 3:**
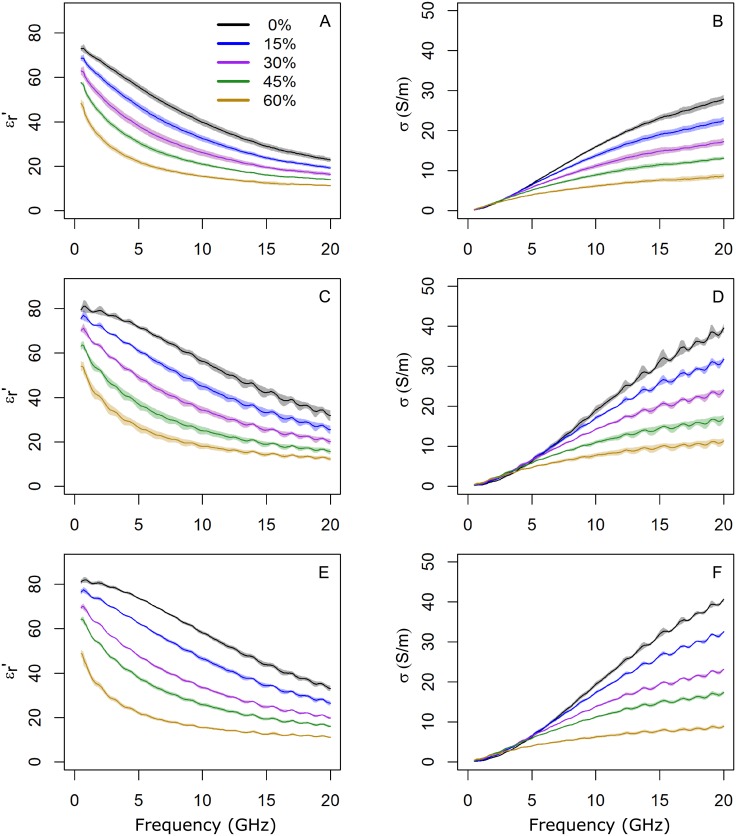
Measurements of the relative permittivity. Average values of the real part of the relative permittivity ($\varepsilon_{r}^{{}^{\prime }}$) and conductivity (*σ*) versus frequency depending on the sucrose concentration, for PVA-C (A,B), agar (C,D) and deionized water (E,F), respectively. The curves are color-coded depending on the sucrose concentration as seen in the legend. The shaded area indicates the magnitude of the corresponding standard deviation.

Fig 3 displays the experimental real part of the relative permittivity and the conductivity, according to sucrose concentration, for all the phantoms. Each curve corresponds to the mean value and the standard deviation for all the data collected daily in a period of 18 days. An increment in sucrose concentration produces a decrement in both the dielectric constant and the conductivity. The relative permittivity follows the same trend for all the designed phantoms at most sucrose concentrations, except at 0% in which the PVA-C-based phantoms present a different behaviour as compared to the deionized water and the agar-based ones. These results are in agreement with previous reports [1, 8, 20, 31]. The addition of sucrose leads to a faster decrement in the dielectric constant with frequency as compared to that of the deionized water [8]. In fact, as can be seen in Fig 3, agar produces a faster reduction of the dielectric constant as compared with deionized water, but a larger effect is noticed for the PVA-C. In addition, a change in the shape of the curves (from concave to convex) was also reported earlier [8]. This effect is observed for agar-based and deionized water phantoms. However, PVA-C-based phantoms do not exhibit this trend.

As can be appreciated, at low frequencies, all models present a linear behaviour for the conductivity and a clear distinction based on the sucrose concentration is not noticed. For higher frequencies, conductivity increases with frequency depending on sucrose concentration and phantom type. Furthermore, the deionized water and the agar phantoms follow the same trend with a pronounced increment as opposed to the PVA-C ones which increases in a more gradual way.

As explained in the previous section, a parameterization of these curves was performed in order to express them as a function of the frequency, *f*, and the sucrose concentration, *S*_*c*_, for each set of phantoms (PVA-C-, agar-based and deionized water). In order to assess the goodness-of-fit for the complex permittivity versus frequency, the pseudo coefficient of determination (*R*^2^) for the real part presented the lowest value for a sucrose concentration of 60%, being 0.984, 0.990 and 0.983 for the PVA-C-, agar-based and deionized water phantoms, respectively. Regarding the imaginary part, the lowest pseudo *R*^2^ was also found for a sucrose concentration of 60% for the agar-based and deionized water phantoms, being 0.998 and 0.997, respectively. For the PVA-C-based phantom, pseudo *R*^2^ presented the lowest value, 0.999, for a sucrose concentration of 0%. Subsequently, the parameters were extracted for the real and imaginary parts of the relative permittivity and are presented in Tables 1 and 2, respectively. Regarding the fitting procedure focused on the sucrose concentration, the lowest pseudo *R*^2^ for the real part of the permittivity was found for the deionized water phantoms, being 0.993, 0.993, 0.988 and 0.985, for *A*_*ε*′_, *B*_*ε*′_, *C*_*ε*′_, and *D*_*ε*′_, respectively. A similar trend was observed for the imaginary part, in which the lowest pseudo *R*^2^ was found for the deionized water phantom, being 0.996, 0.996, 0.983, for *A*_*ε*″_, *B*_*ε*″_, and *C*_*ε*″_, respectively. For the parameter *D*_*ε*″_, the lowest pseudo *R*^2^ in the imaginary part was observed for the agar-based phantom, being 0.992.

**Table 1:** Parameters to obtain the real part of the relative permittivity ($\varepsilon_{r}^{{}^{\prime }}$) according to the sucrose concentration for each gelling agent used.

Parameters	PVA-C	Agar	Deionized Water
$A_{\varepsilon^{{}^{\prime }}}^{{}^{\prime }}$	2.435⋅10^−6^	1.509⋅10^−6^	2.074⋅10^−6^
$A_{\varepsilon^{{}^{\prime }}}^{{}^{\prime \prime }}$	-3.500⋅10^−4^	-4.185⋅10^−4^	-4.640⋅10^−4^
$A_{\varepsilon^{{}^{\prime }}}^{{}^{\prime \prime \prime }}$	3.200⋅10^−4^	6.747⋅10^−3^	7.626⋅10^−3^
$B_{\varepsilon^{{}^{\prime }}}^{{}^{\prime }}$	-1.080⋅10^−4^	-9.800⋅10^−5^	-1.280⋅10^−4^
$B_{\varepsilon^{{}^{\prime }}}^{{}^{\prime \prime }}$	0.013	1.817⋅10^−2^	2.027⋅10^−2^
$B_{\varepsilon^{{}^{\prime }}}^{{}^{\prime \prime \prime }}$	8.670⋅10^−2^	-1.974⋅10^−1^	-2.335⋅10^−1^
$C_{\varepsilon^{{}^{\prime }}}^{{}^{\prime }}$	1.450⋅10^−3^	1.793⋅10^−3^	2.319⋅10^−3^
$C_{\varepsilon^{{}^{\prime }}}^{{}^{\prime \prime }}$	-0.127	-0.218	-0.247
$C_{\varepsilon^{{}^{\prime }}}^{{}^{\prime \prime \prime }}$	-4.496	-1.170	-0.781
$D_{\varepsilon^{{}^{\prime }}}^{{}^{\prime }}$	-4.500⋅10^−3^	-6.584⋅10^−3^	-0.010
$D_{\varepsilon^{{}^{\prime }}}^{{}^{\prime \prime }}$	-0.219	-0.067	0.035
$D_{\varepsilon^{{}^{\prime }}}^{{}^{\prime \prime \prime }}$	75.842	81.730	82.293

**Table 2:** Parameters to obtain the imaginary part of the relative permittivity ($\varepsilon_{r}^{{}^{\prime \prime }}$) according to the sucrose concentration for each gelling agent used.

Parameters	PVA-C	Agar	Deionized Water
$A_{\varepsilon^{{}^{\prime \prime }}}^{{}^{\prime }}$	-2.873⋅10^−6^	-5.752⋅10^−6^	-6.827⋅10^−6^
$A_{\varepsilon^{{}^{\prime \prime }}}^{{}^{\prime \prime }}$	5.501⋅10^−5^	3.410⋅10^−4^	3.648⋅10^−4^
$A_{\varepsilon^{{}^{\prime \prime }}}^{{}^{\prime \prime \prime }}$	8.390⋅10^−3^	2.334⋅10^−3^	3.136⋅10^−3^
$B_{\varepsilon^{{}^{\prime \prime }}}^{{}^{\prime }}$	9.117⋅10^−5^	1.992⋅10^−4^	2.376⋅10^−4^
$B_{\varepsilon^{{}^{\prime \prime }}}^{{}^{\prime \prime }}$	4.455⋅10^−4^	-9.228⋅10^−3^	-9.890⋅10^−3^
$B_{\varepsilon^{{}^{\prime \prime }}}^{{}^{\prime \prime \prime }}$	-0.386	-0.224	0.259
$C_{\varepsilon^{{}^{\prime \prime }}}^{{}^{\prime }}$	-6.193⋅10^−4^	-1.667⋅10^−3^	-2.016⋅10^−3^
$C_{\varepsilon^{{}^{\prime \prime }}}^{{}^{\prime \prime }}$	-0.057	1.362⋅10^−2^	1.358⋅10^−2^
$C_{\varepsilon^{{}^{\prime \prime }}}^{{}^{\prime \prime \prime }}$	5.357	5.162	5.705
$D_{\varepsilon^{{}^{\prime \prime }}}^{{}^{\prime }}$	-7.810⋅10^−4^	3.305⋅10^−4^	-4.311⋅10^−5^
$D_{\varepsilon^{{}^{\prime \prime }}}^{{}^{\prime \prime }}$	0.226	2.448⋅10^−1^	0.306
$D_{\varepsilon^{{}^{\prime \prime }}}^{{}^{\prime \prime \prime }}$	5.746	2.392	0.185

### Cole-Cole model

The wideband dielectric properties data at each sucrose concentration were fitted to a single-pole Cole-Cole for each set of phantoms (PVA-C, agar, and deionized water). The fitting procedure was performed in RStudio [32] using the nonlinear equation system estimation (nlsystemfit) with an ordinary least squares (OLS) algorithm. The parameters (*ε*_∞_, Δ*ε*, *τ*, *σ*_*s*_ and *α*) were limited within physical range to provide a better fit to the experimental data.

Subsequently, each of these parameters was fitted to a suitable polynomial function, according to the sucrose concentration (*S*_*c*_), trading off the minimization of the error fit and the avoidance of overfitting. The same order polynomial fitting, ranging from second to fourth grade, was used for each parameter, being the fourth order polynomial required for the most critical parameters of the model (*τ* and *α*). For the PVA-C-based phantoms, the lowest pseudo *R*^2^ was found for the Δ*ε* parameter, being 0.996. Regarding the agar-based and deionized water phantoms, the lowest pseudo *R*^2^ was observed for the parameter *σ*_*s*_, being 0.927 and 0.993, respectively. Thus, the expressions for each Cole-Cole parameter are listed in Tables 3, 4, and 5 for the PVA-C-, agar-based and deionized water phantoms, respectively.

**Table 3:** Cole-Cole parameters dependent on the sucrose concentration for the PVA-C-based phantoms.

Parameters	PVA-C
*ε*_∞_	-1.698·$10^{-5}\cdot S_{c}^{3}$ + 0.197·$10^{-2}\cdot S_{c}^{2}$ − 2.812⋅10^−2^⋅*S*_*c*_ + 4.273
Δ*ε*	-1.414·$10^{-3}\cdot S_{c}^{2}$ -2.119⋅10^−1^⋅*S*_*c*_ + 68.790
*τ*	7.852·$10^{-6}\cdot S_{c}^{4}$ − 5.842·$10^{-4}\cdot S_{c}^{3}$ + 2.085·$10^{-2}\cdot S_{c}^{2}$ + 8.112⋅10^−2^⋅*S*_*c*_ + 15.340
*σ*_*s*_	3.062·$10^{-7}\cdot S_{c}^{3}$ − 2.121·$10^{-5}\cdot S_{c}^{2}$ − 6.232·$10^{-4}\cdot S_{c}^{2}$ + 1.084⋅10^−1^
*α*	1.671·$10^{-8}\cdot S_{c}^{4}$ − 2.067·$10^{-6}\cdot S_{c}^{3}$ + 5.957·$10^{-5}\cdot S_{c}^{2}$ + 3.042⋅10^−3^ ⋅ *S*_*c*_ + 1.158⋅10^−1^

**Table 4:** Cole-Cole parameters dependent on the sucrose concentration for the agar-based phantoms.

Parameters	Agar
*ε*_∞_	- 3.525·$10^{-5}\cdot S_{c}^{3}$ + 4.864·$10^{-3}\cdot S_{c}^{2}$ − 1.607⋅10^−1^ ⋅ *S*_*c*_ + 5.678
Δ*ε*	-3.109·$10^{-3}\cdot S_{c}^{2}$-1.433⋅10^−1^ ⋅ *S*_*c*_ + 74.280
*τ*	1.156·$10^{-6}\cdot S_{c}^{4}$ + 6.262·$10^{-5}\cdot S_{c}^{3}$ + 1.169·$10^{-3}\cdot S_{c}^{2}$ + 1.414⋅10^−1^ ⋅ *S*_*c*_ + 10.650
*σ*_*s*_	1.580·$10^{-7}\cdot S_{c}^{3}$ -1.076·$10^{-5}\cdot S_{c}^{2}$ + 3.349⋅10^−5^ ⋅ *S*_*c*_ + 1.676⋅10^−1^
*α*	1.276·$10^{-8}\cdot S_{c}^{4}$ − 1.790·$10^{-6}\cdot S_{c}^{3}$ + 4.380·$10^{-5}\cdot S_{c}^{2}$ + 5.283⋅10^−3^ ⋅ *S*_*c*_ + 0.011

**Table 5:** Cole-Cole parameters dependent on the sucrose concentration for the deionized water phantoms.

Parameters	Water
*ε*_∞_	- 2.766·$10^{-5}\cdot S_{c}^{3}$ + 4.551·$10^{-3}\cdot S_{c}^{2}$ − 1.669⋅10^−1^ ⋅ *S*_*c*_ + 5.813
Δ*ε*	-4.724·$10^{-3}\cdot S_{c}^{2}$ -1.127⋅10^−1^ ⋅ *S*_*c*_ + 75.410
*τ*	2.461·$10^{-5}\cdot S_{c}^{4}$ − 2.247·$10^{-3}\cdot S_{c}^{3}$ + 7.112·$10^{-2}\cdot S_{c}^{2}$ − 4.845⋅10^−1^ ⋅ *S*_*c*_ + 10.510
*σ*_*s*_	6.198·$10^{-7}\cdot S_{c}^{3}$ -7.495·$10^{-5}\cdot S_{c}^{2}$ + 3.398⋅10^−3^ ⋅ *S*_*c*_ + 7.578⋅10^−2^
*α*	9.786·$10^{-8}\cdot S_{c}^{4}$ − 1.165·$10^{-5}\cdot S_{c}^{3}$ + 0.388·$10^{-3}\cdot S_{c}^{2}$ + 2.165⋅10^−3^ ⋅ *S*_*c*_ + 0.033⋅10^−1^

**Fig 4:**
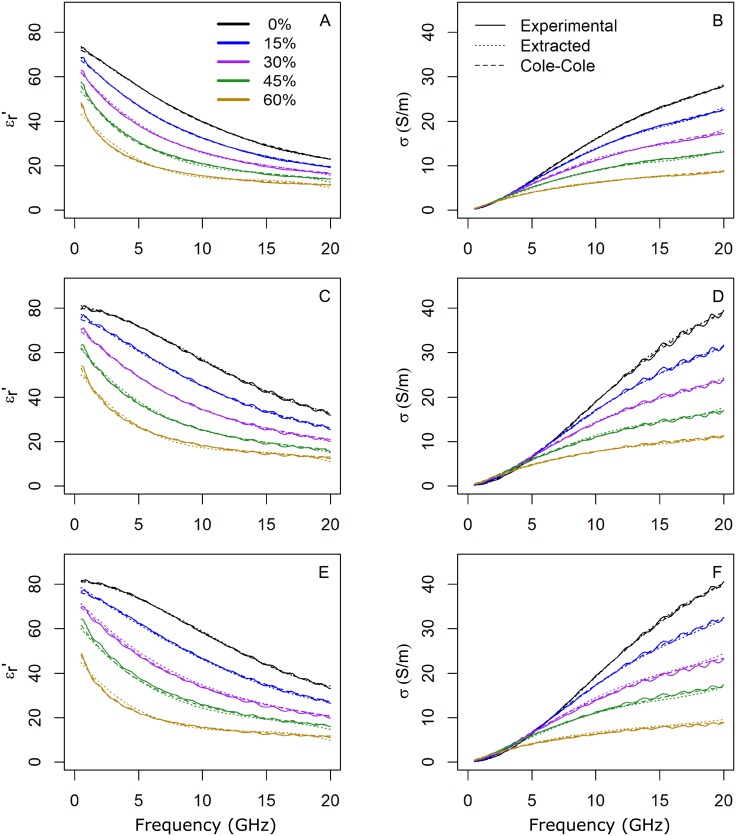
Models fitting. Average values of the real part of the relative permittivity ($\varepsilon_{r}^{{}^{\prime }}$) and conductivity (*σ*) versus frequency depending on the sucrose concentration, for PVA-C (A,B), agar (C,D) and deionized water (E,F), respectively. The curves are color-coded depending on the sucrose concentration as seen in the legend. The solid lines represent the experimental data, the dotted lines represent the extracted model while the dashed lines correspond to the Cole-Cole model.

Fig 4 displays the real part of the relative permittivity and the conductivity, according to sucrose concentration, for each set of phantoms. For each concentration, the experimental data (solid lines), the extracted (dotted lines) and the Cole-Cole (dashed lines) models are shown. As can be seen, for the real part of the relative permittivity, a good agreement is achieved between the experimental data and both models. These behave slightly different at low frequencies (< 1 GHz) which may be due to the trade-off in the fitting procedure. Regarding the conductivity, the models and the experimental data are also in good agreement for all the frequency band. The extracted model has demonstrated similar performance to the theoretical Cole-Cole model.

For water-based tissues within the human body, a correct measurement of the dielectric properties above 10 GHz is hindered due to the relaxation phenomena of water and the penetration depth in tissues above this frequency [33, 34]. However, the proposed models are still valid in the whole frequency range up to 20 GHz. Below 10 GHz, the theoretical values for $\varepsilon_{r}^{{}^{\prime }}$ and *σ* exhibited by biological tissues [35] can be properly mimicked with both gelling agents.

These results indicate that the PVA-C provides the same capabilities as agar as gelling agent, thus validating the extracted model. However, PVA-C presents enhanced viscoelastic properties as compared to agar [36–38], providing better capabilities for multilayer phantom fabrication.

### Model validation

In order to fabricate a phantom that simulates a selected tissue, the value of the real part of the permittivity should be determined [35] and introduced in Eq (3) with the corresponding parameters given in Table 1 for PVA-C- and agar-based phantoms, respectively. By solving this equation, the sucrose concentration required to manufacture the phantom is provided. As a demonstration, Table 6 lists the sucrose concentration required to simulate muscle tissue at different frequencies and the corresponding complex permittivities. These considered frequency values were selected to match common operation frequencies of microwave systems for medical applications [34]. Subsequently, for a targeted frequency and sucrose concentration, the resulted $\varepsilon_{r}^{{}^{\prime }}$ and $\varepsilon_{r}^{{}^{\prime \prime }}$ for the Cole-Cole model were extracted using Eq (6) and the parameters in Tables 3 and 4. $\varepsilon_{r}^{{}^{\prime }}$ and $\varepsilon_{r}^{{}^{\prime \prime }}$ values for the extracted and the Cole-Cole models are presented in Tables 7 and 8 for PVA-C- and agar-based phantoms, respectively. Since depending on the gelling agent, different concentrations are expected for the same permittivity value. It must be noticed that the concentration value provided by the extracted model for the PVA-C was employed to calculate $\varepsilon_{r}^{{}^{\prime }}$ and $\varepsilon_{r}^{{}^{\prime \prime }}$. Therefore, a slightly worse performance was expected for the Cole-Cole model.

**Table 6:** Sucrose concentration, *S*_*c*_(%), required and permittivities, $\varepsilon_{r}^{{}^{\prime }}$, obtained at different frequencies to simulate muscle tissue according to Eq (3).

Frequency (GHz)	$\varepsilon_{r}^{{}^{\prime }}$ [35]	PVA-C *S*_*c*_(%)	PVA-C $\varepsilon_{r}^{{}^{\prime }}$	Agar *S*_*c*_(%)	Agar $\varepsilon_{r}^{{}^{\prime }}$
1	54.811	36.965	54.8110	49.949	54.8108
1.5	53.963	32.957	53.9628	46.785	53.9631
2	53.290	28.917	53.2900	43.647	53.2900
2.5	52.668	25.106	52.6662	40.707	52.6677
3	52.058	21.600	52.0582	38.014	52.0580

**Table 7:** Comparison between the theoretical [35] and the modelled permittivities for muscle tissue at 2 GHz based on a fixed sucrose concentration of *S*_*c*_ = 28.917% for the PVA-C-based phantoms.

*f (GHz)*	$\varepsilon_{r}^{{}^{\prime }}$ *	*σ*(*S*/*m*) *	Extracted		Cole-Cole	
			$\varepsilon_{r}^{{}^{\prime }}$	*σ*	$\varepsilon_{r}^{{}^{\prime }}$	*σ*
1	54.811	0.978	59.162	0.808	59.465	0.529
1.5	53.963	1.188	56.140	1.315	57.093	1.073
2	53.290	1.454	53.289	1.875	54.526	1.728
2.5	52.668	1.773	50.606	2.479	51.893	2.452
3	52.058	2.142	48.082	3.118	49.287	3.210

* Theoretical values for muscle tissue obtained from [35]

**Table 8:** Comparison between the theoretical [35] and the modelled permittivities for muscle tissue at 2 GHz based on a fixed sucrose concentration of *S*_*c*_ = 43.647% for the agar-based phantoms.

*f (GHz)*	$\varepsilon_{r}^{{}^{\prime }}$ *	*σ*(*S*/*m*) *	Extracted		Cole-Cole	
			$\varepsilon_{r}^{{}^{\prime }}$	*σ*	$\varepsilon_{r}^{{}^{\prime }}$	*σ*
1	54.811	0.978	59.379	0.892	60.070	0.571
1.5	53.963	1.188	56.242	1.421	57.401	1.147
2	53.290	1.454	53.290	1.994	54.545	1.832
2.5	52.668	1.773	50.516	2.601	51.655	2.577
3	52.058	2.142	47.914	3.235	48.835	3.346

* Theoretical values for muscle tissue obtained from [35]

The results of this procedure show that the relative permittivity values for a fixed frequency, in this case 2 GHz, are in good agreement with the theoretical value for the extracted model. The observed relative error is below 3% for the PVA-C- and the agar-based phantoms, which is consistent with other reported studies [3, 8]. For frequency values in the interval between 1 and 3 GHz, the models present a moderate relative error being still less than 10% of the theoretical value. Regarding the gelling agent, the agar phantoms present a vaguely larger deviation.

It is possible to design the phantom targeting the imaginary part of the permittivity instead of the real one. However, as can be noticed, none of the models perform particularly well regarding conductivity values, which are higher than the theoretical ones. This trend has been observed in the literature for water-based [8] as well as acetonitrile-based phantoms [9]. This effect may be due to the nominal conductivity of the deionized water, which inherently limits the range of dielectric properties, particularly at high frequencies [3]. Previously, NaCl has been commonly used to control the conductivity of the mimicked tissues, raising to higher values when increasing NaCl concentration [39, 40]. NaCl may provide a good match to the theoretical conductivity of the mimicked tissues. However, the phantom’s conductivity is affected by the nominal conductivity of the water, independently of the gelling agent used or NaCl concentration added, differing considerably at high frequencies [3, 41]. Further work is required to precisely assess the optimum conductivity values for the mimicked tissues.

Regarding the shelf-life of the fabricated phantoms, the consistency of the agar-based phantoms has changed, after seven months storage in a controlled environment (4°C) without being used, becoming more aqueous and exuding water [7]. In addition, some deionized water phantoms have started to develop fungi. The dielectric properties of PVA-C-, agar- and deionized water phantoms, in which mould has not been detected, were measured. The dielectric properties have not varied significantly for the PVA-C- and agar-based measured phantoms, being the average coefficient of variation within 3% and 4% for $\varepsilon_{r}^{{}^{\prime }}$ and $\varepsilon_{r}^{{}^{\prime \prime }}$, respectively. For the measured deionized water phantoms, the corresponding average coefficient of variation was within 2% and 3%.

### Multilayer phantoms

A two-layer phantom was fabricated to demonstrate the suitability of PVA-C as gelling agent for multilayer phantoms. Each layer consisted of approximately 3 cm and contained a varied, randomly-chosen concentration of sucrose, being 0% and 20%, the upper and bottom layer, respectively. Both layers received a simultaneous single freeze and thaw cycle during the fabrication procedure. The homogeneity of the dielectric properties was ensured by measuring complex permittivity at five random spots averaging ten consecutive acquisitions in each spot. These spots included areas in the near proximity of the interface between both layers as well as a couple of centimetres apart in each and opposite direction. It must be noted that the diameter of the measuring probe is approximately one centimeter.

The mean percentages of variation for the measured permittivities were approximately within 0.5% and 2% for the upper and the bottom layers, respectively. Regarding the conductivity, the corresponding values were within 1% and 1.5%. These values are within the range of uncertainty previously reported for the system. Geometric stability is maintained, with a clear interface between layers and no observable changes in the dielectric properties, indicating negligible diffusion of materials.

## Conclusion

The use of an alternative gelling material, named polyvinyl alcohol cryogel (PVA-C) is proposed for the fabrication of custom phantoms in order to characterize microwave systems in a large frequency band (from 500 MHz to 20 GHz). As shown, PVA-C provides the same capabilities as agar as gelling agent, but replicating the elasticity and viscosity of soft tissues with an improved strength and shelf-life. In addition, this polymer keeps its wetness for a longer period of time. Furthermore, PVA-C provides the means to develop multilayer and more realistic complex phantoms, which is obviously not possible with liquid phantoms.

Mathematical models have been developed in which the dielectric properties are parameterized as a function of frequency and sucrose concentration. These models show a good agreement with theoretical models over a wide frequency range (up to 20 GHz) for the relative permittivity. Thus, they enable researchers to customize phantoms, estimating the dielectric properties of the tissue to be mimicked at any frequency in the previous mentioned range. Future activities will aim to adjust the conductivity of tissues by selecting a suitable number of freeze and thaw cycles.
